# Influence of Narrow Titanium Dental Implant Diameter on Fatigue Behavior: A Comparison between Unitary and Splinted Implants

**DOI:** 10.3390/jcm13061632

**Published:** 2024-03-13

**Authors:** Rodrigo González Terrats, María Bonnín Liñares, Miquel Punset, Meritxell Molmeneu, José Nart Molina, Vanessa Ruíz Magaz, Matteo Albertini, José María Manero, Javier Gil Mur

**Affiliations:** 1 Department of Periodontology, School of Dentistry, Universitat Internacional de Catalunya, Carrer Josep Trueta S/N, Sant Cugat del Vallés, 08195 Barcelona, Spain; maria_bonnin@uic.es (M.B.L.); josenart@uic.es (J.N.M.); vanessaruiz@uic.es (V.R.M.); malbertini@uic.es (M.A.); 2 Biomaterials, Biomechanics and Tissue Engineering Group (BBT), Department of Materials Science and Engineering, Universitat Politècnica de Catalunya (UPC), Av. Eduard Maristany 16, 08019 Barcelona, Spain; miquel.punset@upc.edu (M.P.); meritxell.molmeneu@upc.edu (M.M.); jose.maria.manero@upc.edu (J.M.M.); 3 Barcelona Research Centre in Multiscale Science and Engineering, Technical University of Catalonia (UPC), Av. Eduard Maristany, 10–14, 08019 Barcelona, Spain; 4 Institut de Recerca San Joan de Déu (IRSJD), 08034 Barcelona, Spain; 5 CIBER Centro de Investigación Biomédica en Red de Bioingeniería, Biomateriales y Nanomedicina, Instituto de Salud Carlos II, 28029 Madrid, Spain; 6 Bioengineering Institute of Technology, Facultad de Medicina y Ciencias de la Salud, Universitat Internacional de Catalunya, Sant Cugat del Vallés, 08195 Barcelona, Spain

**Keywords:** narrow dental implant (NDI), unitary implants, two-splinted implants, ISO 14801, wöhler S/N curves, SEM fractography, static and cyclic loading, maximum static fracture strength (Fmax), fatigue limit (LF)

## Abstract

**Background:** Scientific literature lacks strong support for using narrow diameter implants (NDI) in high masticatory force areas, especially in molars. Implant splinting in cases of multiple missing teeth reduces lateral forces, improves force distribution, and minimizes stress on implants. However, no studies have evaluated the fatigue load resistance of unitary or splinted implants. **Methods:** This in vitro study compares five groups of new metal alloy implants, including unitary and splinted implants with varying diameters. Mechanical characterization was assessed using a BIONIX 370 testing machine (MTS, Minneapolis, MN, USA) according to ISO 14801. For each of the five study sample groups, (n = 5) specimens underwent monotonic uniaxial compression at break testing and (n = 15) cyclic loading to determine the maximum force (Fmax) and the fatigue life (LF) values. Scanning electron microscopy (SEM) was employed for the fractographic analysis of the fractured samples. **Results:** The Fmax values for unitary samples ranged from 196 N to 246 N, whereas the two-splinted samples displayed significantly higher values, ranging from 2439 N to 3796 N. Similarly, the LF values for unitary samples ranged from 118 N to 230 N, while the two-splinted samples exhibited notably higher values, ranging from 488 N to 759 N. **Conclusions:** The observed resistance difference between sample groups in terms of Fmax and LF may be due to variations in effective cross-sectional area, determined by implant diameter and number. Additionally, this disparity may indicate a potential stiffening effect resulting from the splinting process. These findings have significant implications for dental clinical practice, suggesting the potential use of splinted sets of small-sized NDI as replacements for posterior dentition (premolars and molars) in cases of alveolar bone ridge deficiencies.

## 1. Introduction

Narrow diameter implants (NDI) have a diameter less than 3.5 mm and are categorized into three groups: category 1 (Ø < 2.5 mm), category 2 (2.5 mm < Ø < 3.3 mm), and category 3 (3.3 mm < Ø < 3.5 mm [1]. In natural dentition, occlusal loads range from 100 N in incisors to approximately 700 N in the second molar area. Recent research [2] confirmed similar findings, with males exhibiting bite forces of 289–295 N for incisors, 389–401 N for premolars, and 654–665 N for molars. Females showed forces of 205–223 N for incisors, 332–345 N for premolars, and 568–570 N for molars.

The use of NDI in areas with higher masticatory force has not been strongly supported, as reported in a classical finite element study, where it was shown that a reduced implant diameter was associated with greater marginal bone strain around the neck of the implant and prosthetic connection, resulting in greater marginal bone loss [3].

Fatigue is the main cause of failure in implant connections. It is defined as progressive crack prolongation resulting in a catastrophic fracture under repeated loading below the yield stress [4,5,6]. Implant fatigue resistance is mainly related to the implant diameter, as stress distribution is influenced by the implant diameter. As the diameter of an implant increases, the resistant cross-section increases as a result. NDIs made of Ti-6Al-4V metal alloy present a higher risk of implant fracture when under occlusal loads relative to the premolar-molar area of a human adult [7,8]. However, with the introduction of new metal alloys such as titanium-zirconium alloys (Roxolid^®^) or new technologies for the treatment of metal alloys such as a commercially pure titanium at 30% of cold work (OPTIMUM^®^), there is a decrease in the Young’s elastic modulus of the implants, increasing their fatigue resistance [9].

When restoring an edentulous area with multiple missing teeth, splinting implants minimizes the lateral forces on the prosthesis, increasing force distribution and reducing the stress on dental implants [10]. Even though splinting implants with internal connection does not present statistically significant differences in implant survival rate when compared with single implants, a tendency for fewer mechanical complications has been observed [11]. This is why, when splinting NDI in the posterior area, less hardware complications such as veneer ceramic chipping, screw loosening or fracture, framework fracture, implant fracture, and loss of retention are found (15.5% vs. 39.4%) [12].

The mechanical properties of implant/abutment connections have been studied for single-tooth implant replacements [13]. As far as we know, nowadays there are no published studies which evaluate the fatigue load resistance of two-splinted implants when compared with unitary implants. The main objective of this study focuses on evaluating how much both splinting and implant diameter increase the fatigue resistance of dental implants, while simultaneously assessing the suitability of the use of so-called narrow diameter implants both individually and splinted with a second implant as replacements for posterior dentition (premolars and molars) in cases of alveolar bone ridge deficiencies.

## 2. Materials and Methods

### 2.1. Implant and Restoration Characteristics

**Figure 1:**
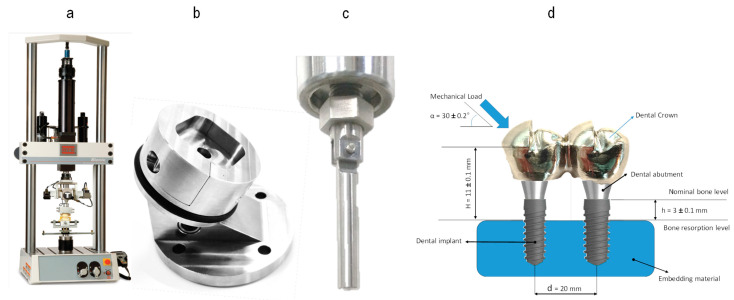
Mechanical testing Set-Up: (**a**) BIONIX 370, (**b**) lower clamping grip, (**c**) upper load grip, and (**d**) schematic diagram of an embedded two-splinted implant sample.

NDIs of 3.0 mm in diameter, 1.85 mm hex diameter, and 10 mm in length, made of Ti GR4, cold worked, and of high tension (OPTIMUM^®^ MicroVega^®^ Klockner^®^ S.A., Escaldes-Engordany, Andorra), and standard diameter implants of 4.0 mm in diameter, 2.35 mm in hex diameter, and 10 mm in length, made of Ti GR4 (OPTIMUM^®^ RegularVega^®^ Klockner^®^ S.A., Barcelona, Spain) were selected. All implant systems used for mechanical testing were assembled according to the surgical protocols. All implants were restored using a 2 mm height abutment (Permanent^®^ Klockner^®^ S.A., Barcelona, Spain). The unitary restorations were restored with a 10 mm mesio-distal spherical tip (cap), simulating the mean mesio-distal distance of a first molar, and the multiple restorations were restored with a 20 mm mesio-distal restoration, simulating the distance of a first and second molar [14] (Figure 1). The crown was selected from the program’s library (Exocad—Archimedes Pro, La Seu d’Urgell, Spain) and was milled on a SAUER 10 five-axis milling machine (Isny im Allgäu, Germany).

### 2.2. Preparation of Specimens

All samples were prepared and conducted in accordance with the ISO-14801 standard [15]. Five different sample groups were established. M1 included unitary 3.0 diameter implants, M2 included unitary 4.0 diameter implants, M3 included two splinted 3.0 diameter implants, M4 included two-splinted 4.0 diameter implants, and M5 included a 3.0 diameter implant splinted to a 4.0 diameter implant, respectively. The rationale for the implant distribution of groups M3, M4, and M5 followed a linear sequential arrangement with respect to the load application axis, placing one implant in front of the other. However, in those two-component splinted sets of different implant diameters, the smaller implant was always placed in front of the larger one to respect the smaller size of the anterior teeth (premolars) in relation to the posterior teeth (molars) and to analyze the splinted set of two implants under the most critical conditions from the mechanical point of view [16,17]. Before mechanical testing, all implants were embedded into a simile to bone polymeric resin (Mecaprex MA2+, PRESI SAS, Eybens, France), leaving them 3 ± 0.1 mm above the theoretical nominal bone level [9,16,18,19,20,21,22,23,24].

### 2.3. Mechanical Characterization

Mechanical evaluation was performed using a servo hydraulic mechanical testing machine BIONIX 370 (MTS, Minneapolis, MN, USA) equipped with a 25 kN load cell and controlled by software Telstar II (version 5.0, Telstar, MTS System Corp, Minneapolis, MN, USA). All tests were conducted at room temperature under dry conditions. Samples (n = 5) from each group were subjected to a monotonic uniaxial compression at break test to determine the maximum breaking load. A custom designed steel holder was used to fix the specimen with a 30° angle of inclination. Load was applied to the distal cusp of the implant-supported restoration in the unitary sample groups, while in the splinted sample groups, it was directed towards the distal cusp of the mesial implant.

Cyclic loading tests were designed at between 10% and 80% of the previously determined Fmax value for each group [16,17,24,25]. Samples (n = 15) per group were used to perform this test, which were loaded under compression-compression cyclic loading at 15 Hz with a load amplitude of R = 0.1. The total number of cycles was fixed at 5 × 10^6^ [9,24]. 

### 2.4. Fractography

The morphology of the fracture was evaluated via scanning electron microscopy (SEM) using the Phenom XL Desktop SEM microscope (PhenomWorld, Eindhoven, Netherland) with an accelerating voltage of 20 keV. The microscope facilitated an X-ray microanalysis using image analysis software (ImageJ, Matlab 3.0 version, Natwick, MA, USA).

## 3. Results

**Figure 2:**
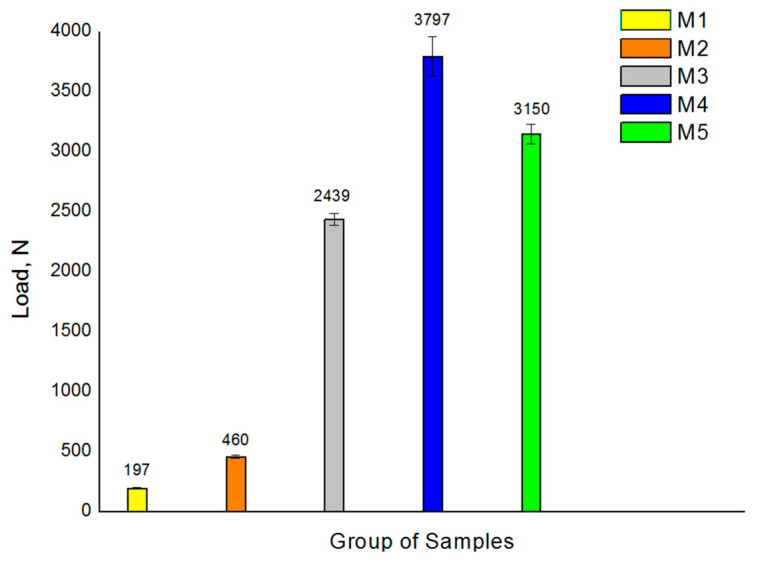
Compression test results for the 5 samples analyzed. Values presented correspond to the mean maximum load at fracture with its standard deviation.

The comparative analysis of maximum static fracture strength (Fmax) values has shown a large increase in the strength of the splinted sets of two implants as compared to the single sets (Figure 2). Although the splinted groups (M3 and M5) have a cross section twice that of the unitary groups (M1 and M3), they have shown maximum breaking strength values between eight (M1 group) and 12 (M3 group) times higher.

Table 1 presents the maximum and minimum loads, the number of cycles endured by each tested sample under their respective load levels, along with the location and description of the specific failure mode observed in each sample. A preliminary examination of the S-N curves showed that higher loads resulted in fracture at a relatively low number of loading cycles in all groups of samples [25]. A more in-depth analysis of the S/N curves made it possible to distinguish three main regions [26]. The first region, the “finite life region”, corresponds to the load range where all specimens showed failure after a finite number of cycles. The second region, the “transition region”, corresponds to the load range where some specimens showed failure, and others withstood the full number of cycles without apparent failure. Finally, the third region, “the infinite region”, corresponds to the load range beyond which all specimens have withstood the total number of fatigue cycles without showing any fracture, also known as “runouts”. T1 = implant and screw rupture, T2 = implant deformation, T3 = screw rupture, T4 = both screws broken but no implant fractured, T5 = 4 mm implant screw broken. Implant not broken, T6 = No break (runout). (*) Indicator of having reached 3 runouts.

The unitary groups M1 and M2 exhibited the lowest fatigue strength due to their smaller cross-sections, the comparison of which has verified that increasing the implant diameter leads to an increase in fatigue strength. Similarly, all two-splinted groups displayed higher mechanical fatigue resistance due to their larger cross-section. Groups M4 and M5 had the largest LF values, followed by sample M3 (Figure 2).

Comparative analysis of the failure modes further revealed disparities among the various groups of samples examined. Notably, the implants in groups M1, M2, and M3 did not exhibit any form of fracture under the load conditions simulating infinite lifespan. However, the M4 and M5 groups displayed screw breakage within one of the two interconnected implants.

**Figure 3:**
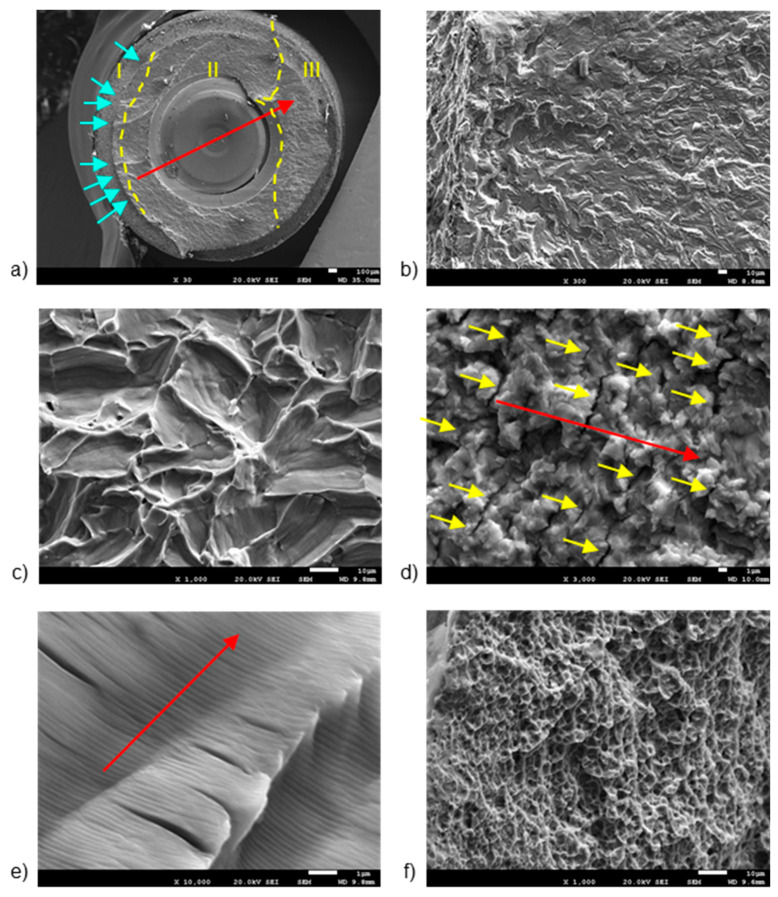
SEM images of fractured implants, detailing: (**a**) fracture regions, (**b**) multiple crack initiation points, (**c**) trans-granular fracture, (**d**) secondary cracking, (**e**) fatigue striations, and (**f**) micro-cavities or “dimples”. The dashed lines are the division of the different fatigue zones. The first one is the crack nucleation (zone I), the middle one is the crack propagation zone (zone II) and the last one is the ductile fracture (zone III). The arrows indicate the crack initiation zone. The scale bars are located at the lower right corner of the images, with values of 1 µm (**d**,**e**), 10 µm (**b**,**c**,**f**), and 100 µm (**a**), respectively.

**Figure 4:**
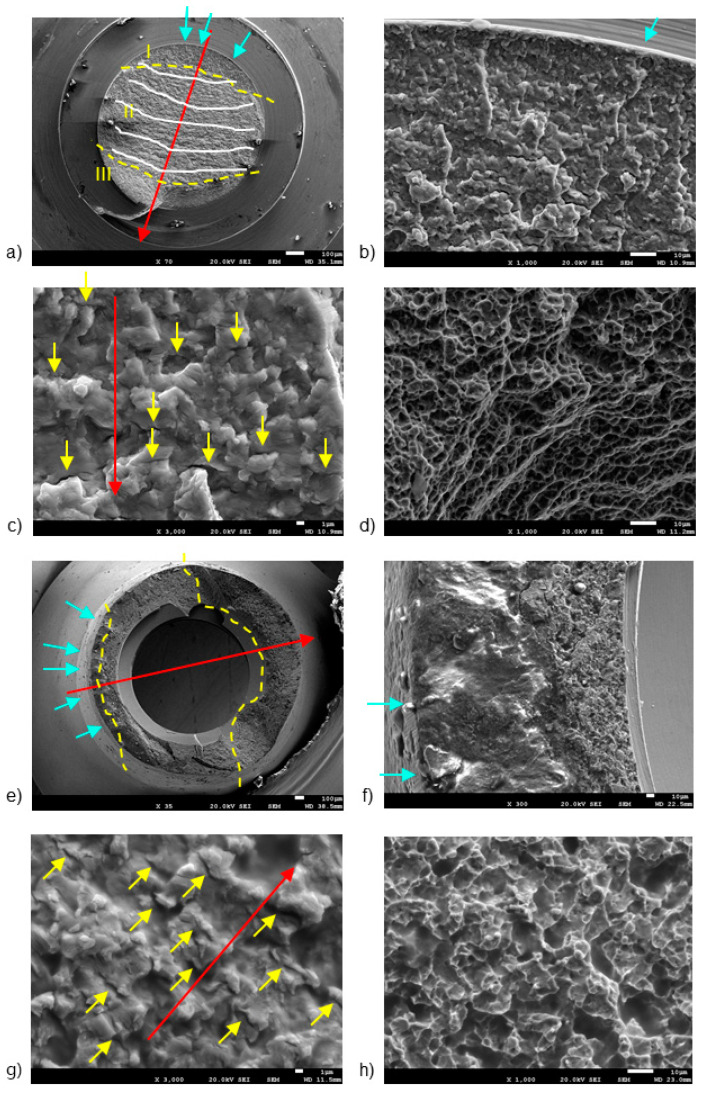
SEM images of fractured screws (**a**–**d**) and abutments (**e**–**h**), detailing: (**a**) fracture regions and benchmarks, (**b**) crack initiation points, (**c**) secondary cracking, (**d**) dimples, (**e**) fracture regions, (**f**) fracture initiation region, (**g**) secondary cracking, and (**h**) intricate micro cavity formation. The dashed lines are the division of the different fatigue zones. The first one is the crack nucleation (zone I), the middle one is the crack propagation zone (zone II) and the last one is the ductile fracture (zone III). The arrows indicate the crack initiation zone. The scale bars are located at the lower right corner of the images, with values of 1 µm (**c**,**g**), 10 µm (**b**,**d**,**f**,**h**), and 100 µm (**a**,**e**), respectively.

Figure 3 and Figure 4 display representative SEM micrographs of the fractured components, namely the implant, as well as the screw and abutment, respectively. Fractographic analysis revealed the following findings: (i) Regardless of the implant diameter or quantity, all fractured implants consistently displayed a fracture plane within the internal threaded hole, just below the final lower plane of the tightening screw. (ii) Fracture planes of the implants exhibited multiple fracture initiations in the upper region, particularly on the rough surface of the thread valleys [27]. (iii) Similarly, all fractured tightening screws exhibited fractures in a consistent plane at the junction between the screw head and the threaded stud, specifically in the lower plane of the screw head. (iv) The fracture planes of the fractured screws also showed fracture initiation in the upper region, specifically on the rough surface of the thread loops.

The first notable feature observed by SEM on the fracture surface was the macroscopically distinct contrast between the initial nucleation crack zone (region I), stable fatigue crack growth zone (region II), and the catastrophic overload fracture zone (region-III) [28,29]. SEM analysis in region II reflected the presence of characteristic cyclic fatigue loading patterns in the form of thin parallel lines commonly referred to as “striations” [27], as well as arrays of parallel micro cracks that were perpendicular to the main direction of fracture propagation, also known as “secondary cracking” [30,31,32]. In the ultimate catastrophic failure region III, the fracture became unstable, leading to an “overload” fracture characterized by significant plastic deformation and “dimple” or micro-cavity formation [28,29,33].

Figure 3 shows the breakage section of a fractured implant, with details of the different fracture zones separated by yellow dashed lines (Figure 3a). Region I shows the presence of multiple crack initiation points (blue arrows in Figure 3b), located on the rough outer surface [27,28]. The directionality of river marks in region I helps pinpoint the fracture initiation point [34,35]. Specifically, the coalescence point of rift marks serves as an indicator for this initiation. SEM analysis of region II enabled the observation of a trans-granular fracture type (Figure 3c), as well as the presence of secondary cracks (Figure 3d) and fatigue striations (Figure 3e). River marks were detected parallel to the direction of fracture propagation (Red arrows in Figure 3b). Region III showed the formation of “dimple” micro-cavities (Figure 3f).

Figure 4 shows the fracture sections of both the broken screw and the abutment, highlighting three distinct fracture regions marked by yellow dashed lines. Region I, situated on the outer surface of both the screw (Figure 4b) and the abutment (Figure 4b), is likely exposed to bending and tensile stresses.

In the screw, region I was situated near the body-head connection zone, specifically within the thread valley, marked by blue arrows [27,36]. Region II had the typical fracture advancement beach marks (indicated by white lines in Figure 4b), as well as secondary crack striations (yellow arrows in Figure 4c). Region III (Figure 4a) displayed notable plastic deformation, leading to the formation of “dimple” micro-cavities (Figure 4d).

In the abutment, region I displayed a few crack initiation points, marked by blue arrows in Figure 4e. Notably, Region I showcased a smooth finish due to the rubbing contact between the crack surfaces under cyclic loading, resulting in a burnished appearance (Figure 4f) [17,37]. Region II displayed characteristic secondary cracks, marked by yellow arrows in Figure 4g.

Crack propagation areas in fractured implants were determined using the ImageJ system. As is well known, crack nucleation is initiated at the surface, and once formed, the mechanical cycles cause crack propagation up to a section where the applied mechanical load exceeds the mechanical strength of the dental implant. Table 2 shows the percentage of propagation area with respect to the total section of the dental implant. It can be seen that the percentage is higher in non-unitary systems, since in the case of two implants, the loads are distributed across the different implants and allow for a longer propagation period.

## 4. Discussion

The findings of this study align with prior research [6,38], indicating that normal and wide diameter implants demonstrate comparable behavior under fatigue loads, which is statistically superior to narrow diameter implants (NDIs), as previously noted [7,8].

All these results are in accordance with the data obtained in our study, where the M2 group presented statistically significant greater LF compared to the M1 group. However, we were able to find the infinite region for both groups (118 N and 230 N). The same phenomenon was observed in the splinted implant groups, where there was a proportional correlation between fatigue resistance and a diameter increase in the splinted implants. We were also able to find the infinite regions of groups M3, M4, and M5 (488 N, 759 N, and 630 N, respectively). Thereby, we can confirm all our stated hypotheses.

When analyzing the results obtained between groups M1 and M2, these are in accordance with previous published articles. In a previous study [10], four different diameter implants (4.0, 3.1, 2.8, and 2.3) were compared under static and cyclic loads. When analyzed, the 4.0 standard diameter implant presented endured a four to ten times greater number of cycles until failure compared with the rest of the NDIs. A recent study comparing implant diameters (2.5 mm, 3 mm, 3.5 mm, and 4 mm) under static load tests found a statistically significant increase in fracture resistance with larger implant diameters [39].

In our study, there was a statistically significant difference between groups M1 (196 N) and M2 (460 N) when the static load test was performed and when a cyclic load was applied (118 N and 240 N respectively), both favoring group M2. We observed that the resistance to fracture in both groups was higher compared to other studies. In a similar study [6], the researchers compared titanium implants alloyed with 15% zirconia and titanium grade 4 implants hardened by 12% cold working with conventional grade 4 titanium implants in NDIs. Both the zirconia-alloyed titanium and cold-worked grade 4 titanium showed better fatigue resistance than conventional grade 4 titanium implants.

Our study shows for the first time that two-splinted implants have been tested under the conditions specified in ISO14801. The results reveal similar behavior in these implants to that observed in the unitary groups. As the diameter increased, so did the fracture resistance under static load and the fatigue resistance under cyclic load, as per conducted tests. In all groups, we observed that during the cyclic load tests, implant and abutment fractures were not observed as the load decreased, and only screw fracture was found until we reached the infinite life region.

When comparing the results obtained between unitary implant groups M1 and M2 and splinted implant groups M3, M4, and M5, a statistically significant increase in fracture and fatigue resistance was observed. When analyzing groups M1 and M3, we found over 10 times greater resistance to fracture when the static load test was performed, and nearly four times greater resistance to fatigue when the cyclic load test was achieved when NDIs were splinted. Furthermore, when an NDI was combined with a standard-diameter implant (comparison between groups M1 and M5), the fracture resistance was over 16 times greater, and the fatigue resistance was nearly five times greater.

These are very interesting results that can help clinicians in daily practice. Forces recorded in the posterior area for a human adult can reach up to 650–700 N [40,41], bearing in mind that this force should be considered as static load. Thus, unitary NDIs can be used in the incisor area, reaching the premolar area in some cases, while splinted NDIs could be used in the posterior area. Splinting NDIs or combining them with standard-diameter implants may be an alternative to bone regeneration in some clinical scenarios.

According to certain authors [42], it is necessary to be cautious with patients exhibiting parafunctional habits, as they indicate that stress distribution increases under parafunctional loads, resulting in greater occlusal loads and escalating the risk for implant fracture.

SEM was used for fractographic analysis of the fractured components. In view of the results obtained, the fracture initiation is found on the external surface of the threaded area of the dental implants. This surface, characterized by a cylindrical self-tapping and rough geometry, would present two main features susceptible to becoming stress concentration points under mechanical solicitation [2]. However, surface modification through shot blasting techniques significantly increased the compression residual tension of the implant surface, which significantly increased the number of cycles until fracture, diminishing the number of stress concentration points and propagation of the initial fracture crack [5,9].

One of the limitations of this study surrounds the study of the influence of mechanical loads on biological behavior. It is well known that mechanical loads favor cellular activity, especially osteoblastic activity, and will therefore favor osseointegration at certain stress levels. Bryniarska-Kubika et al. have published an interesting review [43] where they study the mechanobiology of the cells. This aspect presents a great interest due to providing the principal mechanisms of cells functioning under the contexts of physical stimulation [44,45,46], hydrogel encapsulation [47,48], and substrate stiffness sensing [49,50]. A lot of work in this field has been done so far, mainly in in vitro systems. However, it can be seen that there is a need for more mechanistic research aiming to understand how physical factors contribute to tooth and bone development as well as pain propagation, especially in the process of pulpitis. This future research will provide important knowledge that will allow for the discovery of new therapeutic strategies in the treatment of most bothersome dental diseases.

## 5. Conclusions

The Fmax values for unitary samples ranged from 196 N to 2460 N, whereas two-splinted samples showed significantly higher values, ranging from 2439 N to 3796 N. Similarly, LF values for unitary samples varied from 118 N to 230 N, whereas two-splinted samples displayed notably higher values, ranging from 488 N to 759 N. This disparity is directly associated with the fluctuation in effective cross-sectional area, influenced by both implant diameter and quantity, thus reaffirming the initial hypothesis. The splinting of two implants resulted in greater increases in Fmax and LF values, beyond the theoretical doubling expected based on the resistant section of unitary systems. This suggests that, in addition to the strength gained from the larger resistant section, the fatigue results indicate the presence of a stiffening and/or hardening effect due to the splinting. Hence, the findings acquired from this investigation hold substantial implications for the domain of dental clinical practice, as they introduce the potential application of splinted sets of small-sized dental implants for the replacement of posterior dentition (premolars and molars), as opposed to individual implants with simultaneous guided bone regeneration, in those situations where there is a deficiency in the alveolar bone ridge, which also implies an increase in patient morbidity and economic issues.

## Figures and Tables

**Table 1:** Overall results table of fatigue tests.

Study Group	% Fmax	Fmax (N)	Fmin (N)	N° Cycles to Break (Interval)		Failure Mode
M1	80	157	16	45.348	46.862	T1
	70	138	14	203.994	5 × 10^6^	T1, T2
	65	128	13	1.151.402	5 × 10^6^	T1, T6
	60	118	12	5 × 10^6^ (*)		T6
M2	80	368	37	18.333	23.379	T1
	70	322	32	90.143	156.858	T1
	60	276	28	857.620	5 × 10^6^	T1, T6
	50	230	23	5 × 10^6^ (*)		T6
M3	80	1951	195	1.307	1.501	T1
	50	1220	122	12.153	16.511	T1
	30	732	73	172.893	5 × 10^6^	T1, T3
	20	488	49	5 × 10^6^ (*)		T6
M4	60	2278	228	25.548	36.073	T1
	50	1898	190	55.686	76.174	T1
	40	1519	152	504.919	550.821	T1
	30	1139	114	5 × 10^6^		T4
	20	759	76	5 × 10^6^ (*)		T6
M5	80	2520	252	2.494	3.295	T1
	60	1890	189	21.265	21.447	T1
	40	1260	126	5 × 10^6^		T4
	30	945	95	5 × 10^6^		T4
	20	630	63	5 × 10^6^ (*)		T6

**Table 2:** Percentage of area corresponding to crack propagation. Asterisk and double asterisk indicate statistically significant differences among them (with *p* < 0.001).

Samples	Area Propagation (%)
M1	67.5 ± 8.1 *
M2	69.5 ± 7.3 *
M3	80.1 ± 9.2 **
M4	83.8 ± 7.6 **
M5	81.0 ± 8.1 **

## Data Availability

The data that support the findings of this study are available from the corresponding author upon reasonable request.
